# Genetic analysis of the clusterin gene in pseudoexfoliation syndrome

**Published:** 2008-09-22

**Authors:** Kathryn P. Burdon, Shiwani Sharma, Alex W. Hewitt, Amy E. McMellon, Jie Jin Wang, David A. Mackey, Paul Mitchell, Jamie E. Craig

**Affiliations:** 1 Department of Ophthalmology, Flinders University, Adelaide, Australia; 2 Centre for Eye Research Australia, University of Melbourne, Royal Victorian Eye and Ear Hospital, Melbourne, Australia; 3 Centre for Vision Research, Department of Ophthalmology and Westmead Millennium Institute, University of Sydney, Westmead, Australia; 4 Department of Ophthalmology, Royal Hobart Hospital and University of Tasmania, Hobart, Australia

## Abstract

**Purpose:**

Pseudoexfoliation syndrome is a major risk factor for the development of glaucoma. Following recent reports of a strong association of coding variants in the lysyl oxidase-like 1 (*LOXL1*) gene with this syndrome but low penetrance and variable disease frequency between different populations, we aimed to identify additional genetic factors contributing to the disease. The clusterin (*CLU*) gene has been proposed as a candidate because of the presence of clusterin protein in pseudoexfoliation deposits, its varied levels in aqueous humor of cases compared to controls, and the role of the protein as a molecular chaperone. We investigated the association of genetic variants across *CLU* in pseudoexfoliation syndrome and analyzed molecular characteristics of the encoded protein in ocular tissues.

**Methods:**

The expression of clusterin in relevant ocular tissues was assessed using western blotting. Nine tag single nucleotide polymorphisms (SNPs) across *CLU* were genotyped in 86 cases of pseudoexfoliation syndrome and 2422 controls from the Australian Blue Mountains Eye Study cohort. Each SNP and haplotype was assessed for association with the syndrome.

**Results:**

Clusterin was identified in normal human iris, the ciliary body, lens capsule, optic nerve, and aqueous humor. Post-translational modification gives rise to a 100 kDa precursor protein in ocular tissues, larger than that reported in non-ocular tissues. One *CLU* SNP (rs3087554) was nominally associated with pseudoexfoliation syndrome at the genotypic level (p=0.044), although not when the age of controls was restricted to those over 73 years. Only age and the *LOXL1* diplotype were significant factors in the logistic regression. One haplotype of all nine *CLU* SNPs was also associated (p=0.005), but the significance decreased slightly with the use of the age-restricted controls (p=0.011).

**Conclusions:**

Clusterin is present in ocular anterior segment tissues involved in pseudoexfoliation syndrome. Although one haplotype may contribute in a minor way to genetic risk of pseudoexfoliation syndrome, common variation in this gene is not a major contributor to the risk of pseudoexfoliation syndrome.

## Introduction

Pseudoexfoliation syndrome is characterized by an age-dependent deposition of abnormal fibrillar material in both ocular and non-ocular tissues. The most readily identifiable pathological manifestation is the appearance of this extracellular material on the aqueous bathed surfaces of the anterior segment of the eye, particularly the anterior lens capsule. Pseudoexfoliation syndrome is a common cause of open-angle glaucoma. It has also been associated with cataract, particularly of the lens cortex, as well as increased risk of vitreous loss during cataract extraction [1]. An association with cardiovascular disease has also been reported [2,3].

The prevalence of pseudoexfoliation syndrome is known to vary widely between geographic regions, and risk increases significantly with age. The Reykjavik Eye Study in Iceland reported an overall prevalence of 10.6% in patients over 50 years of age increasing to 40.6% in individuals over 80 years of age [4]. Another population-based study, which was conducted in the Blue Mountains, west of Sydney, Australia, found an overall prevalence of 2.3% in individuals over 49 years [5] while the reported prevalence in Greece is 27% among age-related cataract patients [2]. Formal heritability estimates for this condition have not been calculated. However, the risk to relatives of patients with pseudoexfoliation syndrome was found to be 10 times that of the general population in Norway [6]. Recent genetic studies in multiple populations have convincingly identified the lysyl oxidase-like 1 (*LOXL1*) gene as a significant contributor to the genetic risk of developing pseudoexfoliation syndrome [7-11]. The common alleles of two coding variants in this gene are strongly associated with pseudoexfoliation in multiple populations including the Blue Mountains Eye Study cohort. The risk haplotype (G-G) of these two *LOXL1* variants occurs in the homozygous state at a frequency of approximately 25% in normal individuals over the age of 50 and appears to be the ancestral allele [8]. While the frequency of pseudoexfoliation syndrome in Nordic populations is very high, consistent with this allele providing the majority of the risk, the disease prevalence is much lower in populations such as Australia and North America despite similar gene frequencies of the *LOXL1* variants to those found in Iceland [7-9]. Moreover, in the Japanese population, the T-G haplotype is the most common and confers the greatest risk of disease [11,12]. Taken together, the data suggest that besides the *LOXL1* risk alleles, other genetic variants or environmental factors may contribute to the risk of developing pseudoexfoliation syndrome.

The exact composition of the pseudoexfoliative material is unknown. However, initial work has shown that it consists of a complex glycoprotein-proteoglycan structure [13]. Glycosaminoglycans are a prominent component along with basement membrane proteins including laminin, fibronectin, elastin, and fibrillin [13]. These proteins are produced predominantly by epithelial cells of the iris, lens, and ciliary body [1]. A study by Zenkel and colleagues [14] of differential gene expression between these tissues from eyes with and without pseudoexfoliation syndrome revealed several classes of genes that may be important in the production of pseudoexfoliative deposits. These included genes involved in extracellular matrix metabolism and those related to cellular stress and regulation [14]. Using a proteomics based approach, Ovodenko et al. [15] identified extracellular matrix proteins, tissue metalloproteases and their specific inhibitors, cell adhesion molecules, proteoglycans, complement proteins, and clusterin (CLU - also known as apoliprotein J) as major components of the pseudoexfoliation deposits.

*CLU* is one of the most differentially expressed genes between pseudoexfoliation and normal eyes [14], and clusterin was found to be particularly prominent in pseudoexfoliation material [15]. *CLU* is evolutionarily highly conserved [16]. It encodes a 70–80 kDa primary glycoprotein [17] that is cleaved into α (34–36 kDa) and β (36–39 kDa) subunits. The subunits are linked by disulphide bonds to form functional heterodimers that are secreted from the cell. This glycoprotein is ubiquitously secreted by most cell types and has been identified in most body fluids [18]. Its primary function is to act as an extracellular molecular chaperone, preventing the precipitation and aggregation of misfolded extracellular proteins [18]. Clusterin is a multifunctional protein that can bind lipids, complement factors and membrane, and extracellular matrix proteins. Zenkel and colleagues [19] demonstrated that *CLU* mRNA is found at lower levels in anterior segment tissues of eyes with pseudoexfoliation syndrome than in glaucomatous control eyes by both quantitative reverse transcription polymerase chain reaction (RT–PCR) and in situ hybridization. Immunohistochemistry revealed that in these tissues, clusterin is present primarily in the extracellular space, consistent with its proposed role of preventing the deposition of pseudoexfoliative material [17]. Moreover, lower levels of clusterin are present in the aqueous humor of individuals with pseudoexfoliation syndrome compared to normal individuals [19]. Thus, *CLU* is an attractive candidate genetic factor that may confer individual susceptibility to pseudoexfoliation syndrome. We investigated the hypothesis that common genetic variation in *CLU* could explain the genetic susceptibility of individuals to pseudoexfoliation syndrome. Though the expression of *CLU* mRNA in many eye tissues has been reported, the molecular characteristics of the encoded protein in these tissues are poorly understood. In this study, we also analyzed clusterin in clinically relevant anterior segment tissues by western blotting to determine its characteristics in ocular tissues.

## Methods

### Western blotting

Ocular tissues from post-mortem human eyes were obtained through the Eye Bank of South Australia (Flinders Medical Centre, Adelaide, Australia) and aqueous humor from patients undergoing cataract surgery at Flinders Medical Centre, Adelaide, Australia. All samples were collected following approval of the Human Research Ethics Committee (Flinders Medical Centre, Adelaide, Australia). Human corneas from the Eye Bank eyes are used for transplantation and therefore were not available for analysis. For protein extraction, the iris, ciliary body, lens capsule, and optic nerve were homogenized in 6 M urea, 2% DTT, 2% CHAPS, and 0.1% SDS-containing buffer using the TissueLyser (Qiagen, Doncaster, VIC, Australia). The homogenized lysates were cleared by centrifugation and protein concentration was estimated by the Bradford method [20]. Each protein extract (30 μg) and 20 µl of aqueous humor were size-fractionated on a 12% polyacrylamide gel by SDS–PAGE and transferred onto Hybond C Extra (GE Healthcare, Rydalmere, NSW, Australia). The blot was hybridized with 1:500 dilution of the rabbit anti-CLU primary antibody (Santa Cruz Biotechnology Inc., Santa Cruz, CA) and 1:20,000 dilution of the horseradish peroxidase-conjugated goat anti-rabbit IgG secondary antibody (Rockland Immunochemicals Inc., Gilbertsville, PA). Antibody binding was detected with the ECL Advance Western Blotting Detection Kit (GE Healthcare).

### Subject recruitment

Subjects were recruited from the Blue Mountains Eye Study (BMES), which has been described in detail previously [21]. Briefly, the BMES is a population-based cohort study of individuals aged over 49 years living in the Blue Mountains region, west of Sydney, Australia, and the study is designed to investigate common ocular diseases. The majority of participants are of Northwestern European descent. The study included three main surveys held between 1992 and 2004 and an ancillary survey to include individuals who had moved into the area or had reached the required age between 1998 and 2000. The baseline survey recruited 3,654 participants, 2,564 (70.2%) of whom were re-examined at the 5- and 10-year follow-up surveys. The ancillary study added 1,174 individuals during 1999–2000. The age of participants used in the analyses is at the time of the most recent examination. DNA was extracted from peripheral whole blood obtained at the five-year follow-up surveys. Ethics approval was obtained from the relevant committees of the Westmead Millennium Institute at the University of Sydney (Sydney, Australia), Flinders Medical Centre and Flinders University (Adelaide, Australia). Each participant gave informed consent. This study adhered to the tenets of the Declaration of Helsinki.

Pseudoexfoliation syndrome was specifically examined by slit-lamp as part of a comprehensive ocular examination by an experienced ophthalmologist (P.M.) for all participants. Lens photographs were taken of both eyes for each participant, and these were graded for the presence and sub-type of cataract and other signs including pseudoexfoliation to confirm slit-lamp examination findings. Given the inherent difficulties in detection following cataract surgery, the presence of pseudoexfoliation was deemed not to be classifiable in participants who had undergone cataract surgery (n=334). Analysis was performed comparing the diagnosed pseudoexfoliation cases against both the sub-population where pseudoexfoliation had been clinically excluded (phakic individuals) and the total unselected control population (phakic and pseudophakic individuals). The significance of the results was not affected, and thus the data presented here represent the entire cohort. Furthermore, there were no significant differences in genotype frequencies between the two control groups.

### Genotyping and data analysis

Using the software program Tagger, implented in Haploview 4.0 [22], single nucleotide polymorphisms (SNPs) across *CLU* including the promoter region were selected on the basis of linkage disequilibrium patterns observed in the Caucasian (CEU) samples genotyped as part of the International HapMap Project [23,24]. Nine tagging SNPs, which captured all alleles with an r^2^ of 0.8, were selected. A previous study has shown that this population is a suitable surrogate for the selection of tag SNPs to be used in Australian samples with predominantly Northwestern European descent [25].

Genotyping was performed on 2,508 individuals with the use of iPLEX GOLD chemistry (Sequenom, Inc., Herston, QLD, Australia) on an Autoflex Mass Spectrometer (Sequenom, Inc.) at the Australian Genome Research Facility (Brisbane, QLD, Australia). The SNP name designations given are those used in dbSNP and HapMap. SNP genotyping in control samples was checked for compliance with the Hardy–Weinberg equilibrium. Linkage disequilibrium between markers was calculated using Haploview 4.0 [22].

All analyses were conducted in the full data set as well as by restricting controls to a minimum age of 73 years (range 73–98 years, mean 79.9±5.1 years). This age was not only chosen to retain a significant number of controls (n=1,106) but also to ensure the mean age of controls was older than the mean age of cases (76.4±8.1 years), which would reduce the chance of the control cohort containing “yet to develop cases.” Association analysis of each SNP with pseudoexfoliation was performed using the χ^2^ test implemented in Haploview 4.0 [22] and SPSS (v14.0 SPSS Inc., Chicago, IL). Logistic regression was used to assess the role of *CLU* variants as well as known risk factors for pseudoexfoliation in a multifactorial model in SPSS. The most likely haplotypes of the two *LOXL1* SNPs associated with pseudoexfoliation [7,26] were estimated in HAPLO.STATS [27] for each individual and re-coded to a diplotype (a genotype consisting of two haplotypes). This diplotype was then used as a factor in the analysis along with each *CLU* SNP, age, and sex. All variables were added to the model as a single block. Haplotypes across all nine SNPs in *CLU* for each individual were estimated using the expectation maximization algorithm in HAPLO.STATS, and association with pseudoexfoliation was tested with and without adjustments for the covariates (age, gender, and number of *LOXL1* risk alleles carried [0, 1, or 2]). Analyses were conducted for all nine *CLU* SNPs in a single haplotype as well as for SNPs in linkage disequilibrium blocks.

Power calculations were conducted using the online Genetic Power Calculator [28]. The disease prevalence was set at 2.3% as previously reported in our population [5]. Unselected controls were simulated and the case:control ratio was set at 1:28 to reflect the numbers in this study. The risk allele frequency was varied from 0.2 to 0.4 and always set to the same as that for the marker. Linkage disequilibrium between the marker and the risk allele was set at D’=0.8 or 1.0. The genotype relative risks for the heterozygous/high risk homozygous genotypes were set to 1.5/2.0 and 2.0/3.0 to reflect an additive model.

## Results

### Western blotting

**Figure 1:**
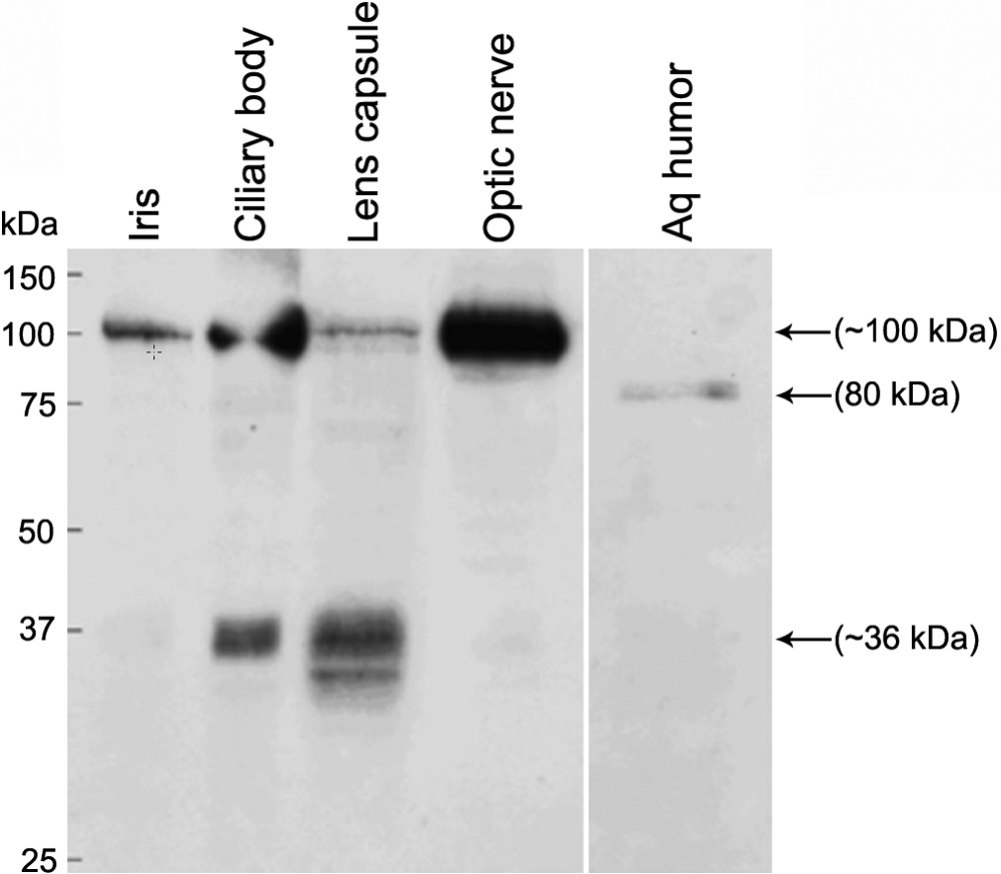
CLU protein expression in human ocular tissues. Expression of the CLU protein in the human iris, ciliary body, lens capsule, optic nerve, and aqueous humor was analyzed by western blotting with an anti-clusterin antibody. Lens capsules from three eyes were pooled for protein extraction. Sizes of molecular weight markers in kiloDaltons (kDa) are indicated. Arrows point to specific protein bands.

We determined CLU protein expression in human ocular tissues of the anterior segment as well as the optic nerve by western blotting under reducing conditions. A protein band of approximately 100 kDa, which is higher than the expected size of the uncleaved primary protein, was detected in the iris, ciliary body, lens capsule, and optic nerve (Figure 1). A prominent protein band of approximately 36 kDa corresponding to the reduced α and β subunits of CLU was identified in the ciliary body and lens capsule. The reduced protein forms were only weakly observed in the iris and optic nerve. A smaller than 36 kDa protein band in the lens capsule may represent a smaller isoform of the α subunit. A protein band of approximately 80 kDa corresponding to the size of the secreted heterodimer was detected in the aqueous humor.

### Genetic analyses

Genotyping was obtained for 2,508 individuals, 57% of whom were male. Pseudoexfoliation syndrome was reported in 86 participants, 63% of whom were male. The mean age of the cohort was 70.1 years with males being slightly older than females (mean±SD; 70.3±10.5 years and 69.8±10.0 years in males and females, respectively), but this was not statistically significant (*t*-test p=0.25). The mean age at examination of pseudoexfoliation cases was 76.4±8.1 years and that of controls was 69.9±10.3 years (p=5.6x10^−9^). The analyses were also conducted by restricting the control group to persons aged 73 years or over (average age 79.9±5.1 years), which is significantly older than the cases (p=1.23x10^−9^). This reduced the number of controls to 1,106.

**Figure 2:**
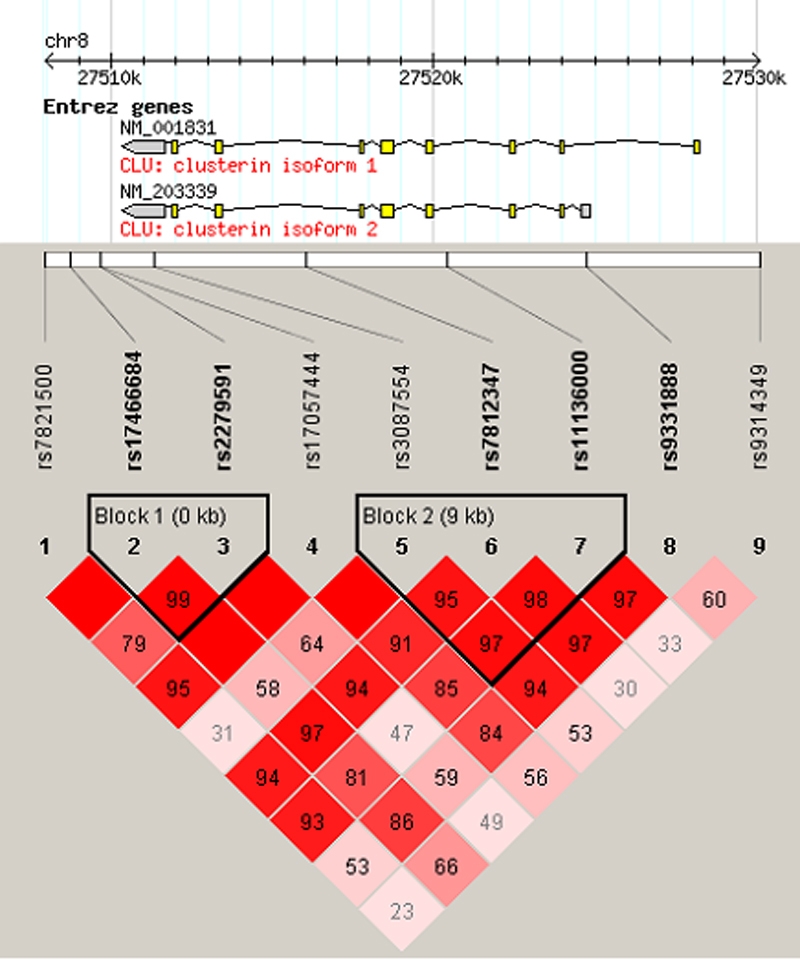
Gene schematic and linkage disequilibrium of genotyped SNPs. The gene schematic is taken from HapMap. Exons are displayed as boxes and introns as connecting lines, and untranslated regions are shaded gray. Linkage disequilibrium structure across *CLU* calculated in Haploview is shown. D′ values are given in the cell intersecting for each pair of SNPs. A blank cell indicates D′=1.0. The darker the cell, the greater the linkage disequilibrium between the SNPs. Haplotype blocks are outlined and were defined using the confidence interval method of Gabriel et al. [29].

All SNPs were in Hardy–Weinberg equilibrium. The linkage disequilibrium between each marker is shown in Figure 2. Using the confidence intervals method of Gabriel et al. [29] incorporated in Haploview 4.0, two haplotype blocks were identified, consistent with the data obtained from HapMap for the original selection of the SNPs from the CEU population, although SNP 8 was also included in block 2 in that data set.

Allele and genotype frequencies along with the p value for the χ^2^ test of independence for allele and genotype counts for each SNP are displayed in Table 1. No significant association was detected at the allele level. A nominally significant genotypic association (p=0.044) observed for SNP rs3087554 did not remain significant following Bonferonni correction (corrected p=0.396). Additionally, this nominal genotypic association was not identified in the age-restricted control group (p=0.072).

**Table 1:** Allele and genotype frequencies for cases (n=86) and controls (n=2422) and p-values for χ^2^ test of independence for allele or genotype counts.

**SNP**	**Name**	**Allele**	**Cases**	**Controls**	**p value**	**Genotype**	**Cases**	**Controls**	**p value**
1	rs7821500	T	0.76	0.69	0.054	T/T	0.56	0.48	0.072
		G	0.24	0.31		T/G	0.40	0.42	
						G/G	0.05	0.10	
2	rs17466684	G	0.83	0.82	0.845	G/G	0.67	0.67	0.848
		A	0.17	0.18		G/A	0.30	0.30	
						A/A	0.02	0.03	
3	rs2279591	C	0.71	0.73	0.560	C/C	0.50	0.53	0.801
		T	0.29	0.27		C/T	0.42	0.40	
						T/T	0.08	0.07	
4	rs17057444	C	0.96	0.95	0.755	C/C	0.93	0.91	0.231
		G	0.04	0.05		C/G	0.06	0.09	
						G/G	0.01	0.00	
5	rs3087554	T	0.84	0.82	0.515	T/T	0.73	0.66	0.044*
		C	0.16	0.18		T/C	0.21	0.31	
						C/C	0.06	0.02	
6	rs7812347	G	0.77	0.72	0.168	G/G	0.58	0.51	0.458
		A	0.23	0.28		G/A	0.37	0.41	
						A/A	0.05	0.08	
7	rs11136000	C	0.53	0.60	0.061	C/C	0.26	0.35	0.242
		T	0.47	0.40		C/T	0.55	0.50	
						T/T	0.20	0.15	
8	rs9331888	C	0.74	0.72	0.445	C/C	0.53	0.51	0.632
		G	0.26	0.28		C/G	0.42	0.41	
						G/G	0.05	0.08	
9	rs9314349	A	0.60	0.61	0.782	A/A	0.34	0.36	0.808
		G	0.40	0.39		A/G	0.52	0.50	
						G/G	0.14	0.14	

The asterisk indicates that p=0.072 when the age of controls is restricted.

Each *CLU* SNP was included in a logistic regression along with the age, gender, and diplotype observed at the *LOXL1* locus. Age and the *LOXL1* diplotype were significantly associated with pseudoexfoliation syndrome as previously reported (p<0.001; Table 2) [7]. No *CLU* SNP was a significant factor in either the full study cohort or in the age-restricted control subgroup. As expected in the full study cohort, the risk of pseudoexfoliation increased with age (OR=1.068; Table 2). However, in the age restricted control group, the direction of association with age was opposite with an increase in age correlating with a decrease in pseudoexfoliation risk (OR=0.886; Table 2).

**Table 2:** Results of logistic regression for the outcome of pseudoexfoliation syndrome for each *CLU* tagging SNP, age, gender, and *LOXL1* diplotype.

**Variables**	**All controls**				**Oldest controls (>73 years)**			
	**p value (Wald test)**	**Odds ratio**	**95% CI for OR**		**p value (Wald test)**	**Odds ratio**	**95% CI for OR**	
			**Lower**	**Upper**			**Lower**	**Upper**
Age	<0.001	1.068	1.043	1.094	<0.001	0.886	0.846	0.927
Sex	0.283	0.779	0.493	1.23	0.204	0.734	0.455	1.183
LOXL1 diplotype	<0.001	1.908	1.504	2.42	<0.001	1.792	1.421	2.259
rs7821500	0.152	1.05	0.982	1.123	0.199	1.047	0.976	1.122
rs17466684	0.923	0.999	0.969	1.029	0.861	1.003	0.971	1.036
rs2279591	0.414	1.018	0.975	1.062	0.396	1.02	0.975	1.067
rs17057444	0.178	1.176	0.929	1.488	0.06	1.324	0.989	1.774
rs3087554	0.338	1.015	0.984	1.047	0.494	1.012	0.979	1.046
rs7812347	0.761	0.994	0.956	1.034	0.916	0.998	0.956	1.041
rs11136000	0.855	1.003	0.97	1.037	0.85	0.997	0.961	1.033
rs9331888	0.483	0.97	0.892	1.056	0.637	0.979	0.894	1.071
rs9314349	0.989	1	0.968	1.032	0.655	0.992	0.96	1.026
Constant	<0.001	0			0.382	0.027		

All variables were added to the model as a block. Significant factors are highlighted in bold. Results are shown for both the whole study and the age-restricted control set.

Haplotype analyses revealed a nominally significant association of haplotype 4 as shown in Table 3 with p=0.005 under the dominant model (OR=1.63, 95% CI 0.81–3.26). This finding remained of borderline significance after Bonferonni correction for the multiple (10) haplotypes considered. It is important to note that the odds ratio and associated confidence interval presented should be interpreted with caution. It is calculated using the estimated haplotype frequencies (based on weighted probabilities of the possible haplotypes for each individual) to infer the theoretical count data for each group. Haplotype 4 is also nominally significant after adjusting for age (p=0.006), indicating that the observed association is independent of this covariate. The significance was further reduced when the age-restricted control set was used (p=0.011), likely due to the loss of power in the reduced sample size. An adjustment for *LOXL1* diplotype did not significantly change this result (data not shown). Haplotype analyses were also performed across a three SNP sliding window, and no significant associations were observed (data not shown). As the linkage disequilibrium structure suggested two haplotype blocks in this region, the haplotype analysis was conducted for each block yet no significant associations were observed (data not shown).

**Table 3:** Haplotype association between variants across the *CLU* gene and pseudoexfoliation syndrome.

	**SNPs**									**Frequency**		**p values**	
**Hap**	**1**	**2**	**3**	**4**	**5**	**6**	**7**	**8**	**9**	**Controls**	**Cases**	**Additive**	**Dominant**
1	T	G	C	C	T	G	T	C	A	0.24	0.28	0.23	0.12
2	G	G	C	C	T	A	C	C	A	0.17	0.15	0.347	0.246
3	T	A	T	C	C	G	C	G	G	0.09	0.08	0.931	0.64
**4**	**T**	**G**	**C**	**C**	**T**	**G**	**T**	**C**	**G**	**0.07**	**0.11**	**0.008**	**0.005**
5	T	A	T	C	T	G	C	G	G	0.04	0.05	0.444	0.42
6	G	G	C	C	T	A	C	C	G	0.04	0.02	0.448	0.453
7	T	G	C	C	T	G	C	C	A	0.03	0.05	0.488	0.458
8	G	G	C	G	T	A	C	C	G	0.03	0.03	0.923	0.971
9	T	G	C	C	T	G	C	G	G	0.03	0.01	0.157	0.156
10	T	G	T	C	T	G	T	C	G	0.02	0.04	0.22	0.22

Haplotypes (Hap) with frequency greater than 2% in the total cohort (n=2508) are shown with the p values for association under additive and dominant models. The nominally associated haplotype 4 is highlighted in bold. SNPs forming the haplotype are numbered as in Table 1.

Power calculations demonstrate that this study had 80% power to detect a reasonable effect size (genotypic relative risk of 2.0 for Aa genotype and 3.0 for AA). As *LOXL1* is known to contribute a significant proportion of the genetic risk of pseudoexfoliation syndrome, it is possible that another genetic modifier locus could have only minor relative risks. The current study design still has power (up to ~60%) at the nominal significance level under a relative risk model of 1.5 in the heterozygote, depending on the frequency of the marker allele (Table 4).

**Table 4:** Power calculations.

**Genotypic relative risk Aa/AA**	**Linkage Disequilibrium D’**	**Risk and marker allele frequency**	**Power at α=0.05**
1.5/2.0	0.8	0.2	0.4
		0.3	0.44
		0.4	0.44
1.5/2.0	1	0.2	0.57
		0.3	0.62
		0.4	0.61
2.0/3.0	0.8	0.2	0.83
		0.3	0.84
		0.4	0.8
2.0/3.0	1	0.2	0.96
		0.3	0.96
		0.4	0.94

Power in this population-based study to detect a significant genetic association for pseudoexfoliation syndrome at the α=0.05 level for different degrees of linkage disequilibrium and allele frequency. Aa=heterozygote, AA=high risk homozygote.

## Discussion

A common haplotype of the *LOXL1* gene has recently been shown to be a major genetic factor associated with pseudoexfoliation syndrome [7]. However, given that the disease-associated haplotype is not fully penetrant, particularly in non-Nordic populations [8,9], and that a different *LOXL1* risk haplotype may be active in Japanese populations [11,12], we hypothesized that other genes are likely to contribute to the risk of developing this disorder. Given its expression in the anterior segment of the eye and the association of the protein with pseudoexfoliation material, *CLU* is a potential genetic factor of susceptibility to pseudoexfoliation syndrome. Different levels of clusterin in aqueous humor of cases when comparing to controls support the hypothesis that genetically determined differences in clusterin expression or stability could contribute to the pathophysiology of pseudoexfoliation.

Expression of *CLU* in the human eye has been previously reported in the cornea, ciliary body, lens, retina, retinal pigment epithelium, and aqueous and vitreous humor [30-33]. In these studies, mRNA expression was detected by RT–PCR and in situ hybridization and protein expression by immunohistochemistry. Protein in aqueous and vitreous humor was revealed by western blotting. The CLU protein undergoes several modifications before the formation of a functional heterodimer. Previous studies were not able to reveal these characteristics of the protein in ocular tissues. Hence, to gain an insight into its various molecular forms, we analyzed CLU protein expression in ocular tissues by western blotting. Consistent with earlier reports, the CLU dimer of expected size was detected in the aqueous humor [33]. This also verified antibody specificity. Presence of the protein in the ciliary body in this study is consistent with its previous immunohistochemical detection in this tissue [33]. Its presence in the lens capsule correlates with it being one of the prominent components of pseudoexfoliation material as identified by proteomics analysis [15]. This is the first report of expression of CLU protein in the human iris and optic nerve. The ciliary body is believed to be the major site of CLU expression in the anterior segment. The present data for the first time reveal that it is also expressed in the human iris and may be secreted into the aqueous humor from this tissue. The molecular mass of the uncleaved primary protein (100 kDa) in the ocular tissues analyzed here (Figure 1) is higher than that detected in non-ocular tissues by others [34]. Tissue specific post-translational modification of the CLU protein can give rise to protein forms of variable molecular masses in different tissues [35-37]. Hence, post-translational modification in ocular tissues may result in a ~100 kDa primary protein. This hypothesis requires further investigation. Furthermore, the western blot data suggest that the majority of the CLU protein in the optic nerve is uncleaved (Figure 1). The biological significance of the predominance of this protein form is as yet unknown.

Allelic and genotypic analyses revealed that common variants in *CLU* and its promoter region do not contribute in a substantial way to the risk of pseudoexfoliation syndrome. The genotype of SNP rs3087554 was nominally associated. However, this association was not significant following Bonferonni correction for the relatively small number of tests or after analysis was restricted to the subset of unaffected controls over 73 years of age. Haplotype 4 was also nominally associated, but the significance was reduced upon restriction to the older controls. This haplotype has a frequency of around 7% in our population and may contribute a small risk of pseudoexfoliation. Haplotype 4 differs only from the most common haplotype 1 at SNP 9 (rs9314349) for which there is no allelic or genotypic association. Thus, there is no obvious consistency between haplotype and single SNP analyses, although these analyses should be considered as complementary because additional information is tested with the haplotypes (i.e. tagging of the causative variant by the combination of alleles). Additionally, logistic regression failed to identify any factors other than age and the *LOXL1* diplotype that were associated with pseudoexfoliation syndrome in this study. Interestingly, the direction of the relationship with age changed when controls were restricted to those over 73 years. This is possibly due to a “healthy survivor” effect and requires further investigation.

The study does not have sufficient power to detect very small genetic effects. This makes it difficult to draw a firm conclusion in relation to SNP rs3087554 and haplotype 4. A weakness of the entire cohort is the difference in age between the cases and controls with the mean age of cases being six years greater than the controls. Therefore, all analyses were also conducted by restricting the age of controls to 73 years or older. This provided a mean age of the controls of 79.9 years, which is older than the cases, and still allowed inclusion of 1,106 controls. Some control participants who could have gone on to develop pseudoexfoliation syndrome as they became older would have been excluded in these analyses, improving homogeneity. However, the power of this test is reduced due to the decrease in the numbers. Our findings will therefore need to be investigated in additional large cohorts.

The methodology used in this study involves the use of “tagging” SNPs. In this approach, the variations selected for genotyping “tag” the known variation in the gene and reduce the amount of genotyping necessary to assess the gene for association. Previous reports have investigated the utility of the Caucasian HapMap data set in Australia and found good correlation [25], indicating that if an association with a common variant exists we would be likely to detect it with this method. This method is particularly adept at detecting pathogenic mutations that fit the “common variant, common disease” hypothesis. However, it is less efficient at detecting multiple rare variants that may have arisen on different genetic backgrounds [38]. SNP rs3087554 could partially tag a functional variant that is not yet included in the HapMap data set from which these SNPs were chosen. In this scenario, the as yet undetermined variant would likely be found on haplotype 4. The SNPs typed in this study cover the immediate 5′ promoter of the gene, but there could be additional upstream or downstream control elements not adequately assessed here. This possibility is important to consider given the reported lower levels of CLU in the aqueous humor of eyes with pseudoexfoliation.

In summary, CLU is present in ocular tissues relevant to pseudoexfoliation syndrome, and others have shown that its titer is reduced in the aqueous humor of eyes with pseudoexfoliation [19]. We demonstrate a previously undocumented expression of CLU in the optic nerve and iris as well as in other ocular tissues relevant to pseudoexfoliation and ocular tissue with specific post-translational modification of the protein. Our data suggest that common variants in this gene are not strong genetic modifiers of the risk of developing pseudoexfoliation in the Australian population. However, one haplotype with a frequency of around 7% may confer some increased risk. Further analysis in other data sets is needed to clarify this. Further work is also required to elucidate which genetic factors in addition to *LOXL1* are responsible for pseudoexfoliation syndrome.
